# Clustering of lung diseases in the family of interstitial lung disease patients

**DOI:** 10.1186/s12890-022-01927-x

**Published:** 2022-04-07

**Authors:** Michelle Terwiel, Jan C. Grutters, Coline H. M. van Moorsel

**Affiliations:** 1 Interstitial Lung Diseases Center of Excellence, Department of Pulmonology, St Antonius Hospital, Nieuwegein, Netherlands; 2 Division of Heart and Lungs, University Medical Center Utrecht, Utrecht, Netherlands

**Keywords:** Family research, Pulmonary fibrosis, Lung diseases, Interstitial

## Abstract

**Background:**

The presence of familial interstitial lung disease (ILD) has been found to predict development of progressive pulmonary fibrosis. However, the role of non-ILD lung diseases in ILD patients’ families has not yet been investigated. We aimed to identify associations between ILDs and non-ILD lung diseases from ILD patients’ self-reported family health history.

**Methods:**

We analysed questionnaires on family health history of 1164 ILD patients for the occurrence of ILD and non-ILD lung disease in relatives. Logistic regression analysis was used to study associations with diagnosis groups.

**Results:**

Familial pulmonary fibrosis was reported by 20% of patients with idiopathic pulmonary fibrosis (IPF; OR 9.2, 95% CI 4.7–17.9), and 15% of patients with unclassifiable pulmonary fibrosis (OR 4.1, 95% CI 2.0–8.2). Familial occurrence was reported by 14% of patients with sarcoidosis (OR 3.3, 95% CI 1.9–5.8). Regarding non-ILD lung disease, significantly more patients with IPF (36%) reported lung cancer in their family (OR 2.3, 95% CI 1.4–3.5), and patients with hypersensitivity pneumonitis (18%) mostly reported COPD (OR 2.3, 95% CI 1.3–4.2). Comparison of sporadic and familial ILD patients’ reports showed that emphysema (OR 4.6, 95% CI 1.8–11.6), and lung cancer (OR 2.4, 95% CI 1.2–4.9) were predictive for familial pulmonary fibrosis, particularly when reported both in a family (OR 16.7, 95% CI 3.2–86.6; *p* < 0.001).

**Conclusions:**

Our findings provide evidence for clustering of ILD and non-ILD lung diseases in families and show that self-reported emphysema and lung cancer of relatives in this population predicts familial pulmonary fibrosis.

**Supplementary Information:**

The online version contains supplementary material available at 10.1186/s12890-022-01927-x.

## Background

Interstitial lung disease (ILD) is a heterogeneous group of pulmonary disorders, which share characteristics in different domains, such as inflammation with or without fibrosis, and clinical symptoms ranging from dyspnea and cough to respiratory failure [1]. The identification of ILD subtypes is crucial for clinical management of disease [2, 3].

Significant familial aggregation of sarcoidosis [4] and of idiopathic pulmonary fibrosis (IPF) [5, 6] has been confirmed in multiple studies. Family health history contains information on both genetic and environmental spheres of risk, and is a powerful tool in risk assessment for common chronic diseases [7, 8]. Moreover, clustering of different diseases provides support for shared disease pathogenesis and shared therapeutic approach. Most studies on the clustering of diseases within families are on autoimmune diseases [9, 10]. We previously reviewed findings on the co-occurrence of sarcoidosis and immune-mediated (chronic inflammatory and autoimmune) diseases in both patients and their relatives [11]. From these studies it is clear that many immune-mediated diseases cluster in the family of patients with sarcoidosis. While several reports suggest significant co-occurrence of ILD and non-ILD lung diseases within patients [2, 3, 12–14], no data is present for familial clustering of ILD and non-ILD lung disease.

Within ILD however, a family history of ILD is now increasingly used to identify subjects at risk for more severe disease in chronic ILDs [15, 16]. Patients with familial pulmonary fibrosis (FPF) appear to have more severe disease evolution and a younger age of onset in comparison to sporadic patients [15, 16], which underlines the need for the identification of these families. Further research on familial pulmonary fibrosis has revealed the existence of monogenic disorders in subgroups of patients with fibrosing ILD [2, 3]. A commonly used way to identify patients with familial pulmonary fibrosis is the self-reported presence of ILD in the family. Studies on this topic included first, second or even up to the 5^th^ degree relatives with IPF only or any confirmed or self-reported ILD [15–21]. However, ILD comprises multiple entities and it is unclear how these diseases cluster in families. Furthermore, it is unknown if non-ILD lung diseases cluster in the families of ILD patients. Therefore, in this study, we aimed to identify clustering patterns of ILDs and non-ILD lung diseases from ILD patients’ self-reported family health history.

## Methods

### Study population

Included in our study were a total of 1358 newly referred patients who were diagnosed with an interstitial lung disease (ILD); who received a questionnaire on family health history of disease in between March 16, 2010 and April 20, 2015. The study was approved by the Medical research Ethics Committees United (MEC-U) of the St Antonius Hospital (R05-08A) and written informed consent was obtained from all participants. Patients were stratified by the ILD diagnosis provided at our ILD outpatient clinic, following recent guidelines by the American Thoracic Society (ATS) and the European Respiratory Society (ERS) [22–28].

Diagnoses of patients were based on medical records and included sarcoidosis (n = 744, including Löfgren’s syndrome); hypersensitivity pneumonitis (HP, n = 102); idiopathic pulmonary fibrosis (IPF, n = 128); unclassifiable pulmonary fibrosis (uPF, n = 126); idiopathic interstitial pneumonia other than IPF (non-IPF IIP, n = 68); autoimmune disease ILD (aidILD, n = 106, including connective tissue disease (CTD) associated-ILD (n = 61), interstitial pneumonia with autoimmune features (n = 24), other (n = 21)); and other interstitial lung disease (oILD, n = 84, including drug-induced interstitial lung disease (n = 21), exposure related ILD (n = 16), lymphangioleiomyomatosis (n = 25), other (n = 24)). Table 1 presents study population characteristics. All patients visiting the ILD outpatient clinic were eligible to submit the form. Demographics age and sex are presented, ethnicity of the patient or family was not noted on the form. Details on the diagnosis types of patients within each diagnosis group are presented in Additional file 2: Table 1.

**Table 1:** ILD study population characteristics

Diagnosis group	Questionnaire provided, N	Questionnaire completed, N (%)	Male, N (%)	Age in years, mean (sd)	Per patient reported relatives with disease, mean (sd)	Per patient reported 1st degree relatives with disease, mean (sd)
Total	1358	1164 (86)	669 (58)*	54.0 (13.6)*	7.1 (5.1)	3.3 (2.2)*
Sarcoidosis	744	644 (87)	368 (57)	47.7 (11.0)	7.1 (5.1)	3.0 (2.0)
HP	102	87 (85)	41 (47)	60.5 (11.4)	7.6 (5.5)	3.9 (2.3)
IPF	128	109 (85)	86 (79)	65.3 (10.3)	6.4 (4.4)	4.0 (2.3)
uPF	126	103 (82)	72 (70)	67.1 (10.1)	6.4 (4.8)	4.0 (2.5)
non-IPF IIP	68	54 (79)	23 (43)	58.3 (13.1)	6.5 (5.0)	3.3 (2.0)
aidILD	106	93 (88)	50 (54)	58.9 (12.4)	7.1 (4.7)	3.6 (2.2)
oILD	84	74 (88)	29 (39)	57.8 (15.4)	8.0 (5.6)	3.6 (2.2)

HP = hypersensitivity pneumonitis; IPF = idiopathic pulmonary fibrosis; uPF = unclassifiable pulmonary fibrosis; non-IPF IIP = idiopathic interstitial pneumonia other than IPF; aidILD = autoimmune ILD; oILD = other ILD. Differences in study population characteristics were tested with ANOVA (post-hoc Scheffe’s) for continues and Chi squared for dichotomous variables; and associations between age and reported first degree relatives with linear regression analysis. *p < 0.05

### Questionnaire on family health history

The questionnaires on history or presence of disease in relatives (Additional file 1) were completed by the patients at first visit. Patients were asked to write down all diseases of relatives known to them. Relative types were systematically ordered on the questionnaire to facilitate patient’s recall, and to enable structured data analysis.

We compared the total of reported families with lung diseases between our ILD patient diagnosis groups. Self-reported diseases in the family included any ILD grouped as: sarcoidosis (including Löfgren’s syndrome reported by 3 patients), pulmonary fibrosis (including two patients who reported ‘stiff lungs’, and another two ‘fibrosis’), and remaining ILD (including reported asbestosis, asbest lungs, asbest lung disease, dust lungs, hypersensitivity pneumonitis, alveolitis, UIP, eosinophil pneumonia, ILD). Non-ILD lung disease reports were grouped as: asthma (reports of asthma or asthmatic), bronchitis (reported bronchitis or asthmatic bronchitis), COPD, emphysema, pneumonia, tuberculosis (including 9 patients who reported ‘pleuritis’), lung cancer, and lung disease not specified (not specified reports of lung and respiratory disease).

Familial ILD was defined as an ILD patient reporting one or more relatives with an ILD. We studied the reports by relative type, that is first degree (parents, siblings, and children), or (only) any other relative. Subsequently, we compared sporadic and familial ILD patients in their reports of ILD and non-ILD lung disease. To investigate differences in pulmonary fibrosis, we combined IPF and uPF data to form a group with familial PF (FPF) and a group with sporadic PF (SPF).

Study data were collected and managed using REDCap electronic data capture tools, hosted at St. Antonius hospital, Nieuwegein, the Netherlands [29, 30].

### Statistical analyses

IBM SPSS Statistics 26 for Windows (IBM Corp, Armonk, NY) was used for statistical analysis. Differences in study population characteristics between the ILD patient diagnosis groups were tested with ANOVA (with Scheffe’s post-hoc test) for continues variables, and Chi-squared test for dichotomous variables. To study if differences in reported first degree relatives were associated with age, we used linear regression analysis. Differences in the proportion of ILD patients from any diagnosis group versus the others in reporting ILD and non-ILD lung disease in their family (one or more relatives with disease in each family) were first assessed with a Chi squared test, or Fisher’s exact when the assumptions were not satisfied. The significance level was set at p = 0.05. For significant results, we used logistic regression to calculate odds ratios for reporting disease in the family, associated with patient diagnosis groups. We also adjusted for sex and age, because these patient characteristics differed between the diagnosis groups, and may influence their reports of disease in relatives. Chi squared, or Fisher’s exact test was used to evaluate if the proportion of patients reporting disease in the family differed between patients with and without familial disease. All significant results are presented with the odds ratio (OR) and 95% confidence interval (CI).

## Results

**Fig. 1:**
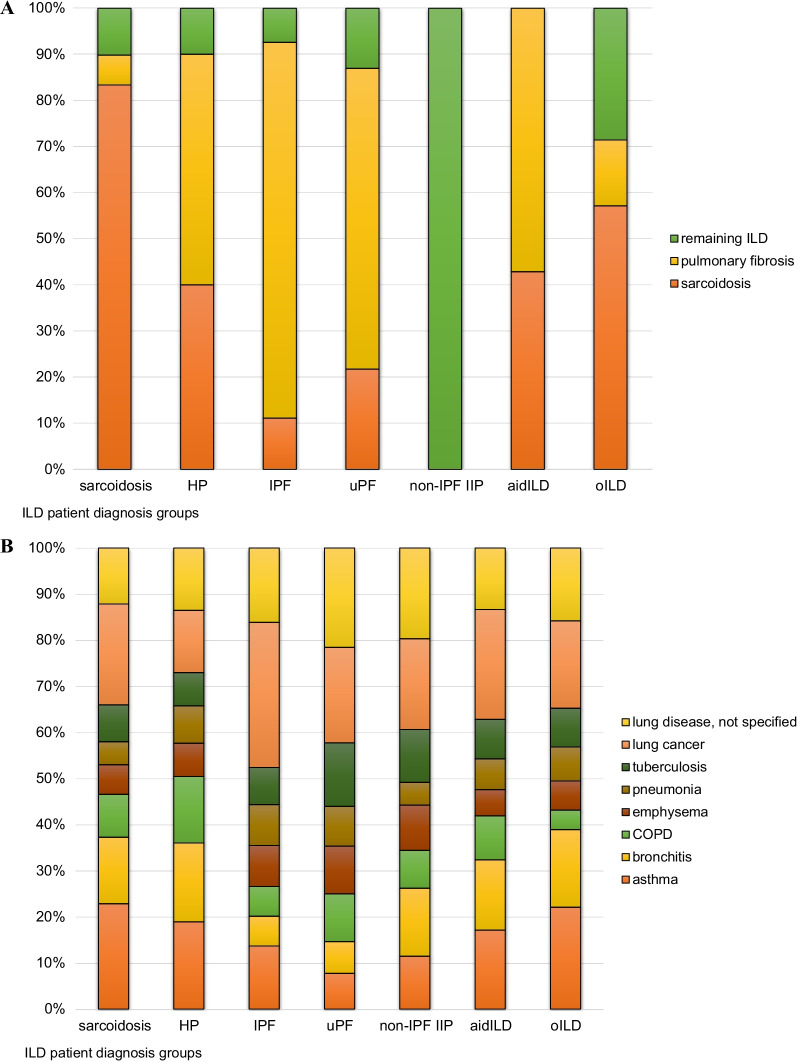
Percentage of patients who reported relatives with ILD (**a**) and non-ILD lung disease (**b**) per diagnosis group. ILD patient diagnosis groups: HP = hypersensitivity pneumonitis; IPF = idiopathic pulmonary fibrosis; uPF = unclassifiable pulmonary fibrosis; non-IPF IIP = idiopathic interstitial pneumonia other than IPF; aidILD = autoimmune disease interstitial lung disease; oILD = other interstitial lung disease

Patients were divided in seven diagnosis groups (Additional file 2: Table 1 for diagnosis types within these groups) with population characteristics presented in Table 1. In total 1164 (86%) out of 1358 interstitial lung disease (ILD) patients completed the questionnaire on family health history, with no significant differences in the percentages of non-responders between the diagnosis groups. Furthermore, there was no significant difference between the patient diagnosis groups in the mean number of total reported relatives. Significant differences included mean age, sex, and the mean number of reported first degree relatives (Table 1). Age was positively associated with the number of first degree relatives reported across all ILD diagnosis groups. Figure 1 shows the proportion of patients reporting ILD (1A) and non-ILD lung disease (1B) in their family (1 or more relatives), per ILD diagnosis group. Numbers and percentages of ILD patients from each diagnosis group, who reported disease in their family are provided in Table 2.

**Table 2:** Number of ILD patients who reported ILD and non-ILD lung disease in their family (1 or more relatives)

Reported disease in family	All	Sarc	HP	IPF	uPF	Non-IPF IIP	aidILD	oILD
N (%)	1164	644	87	109	103	54	93	74
Any ILD or non-ILD lung disease	801 (69)	430 (67)	61 (70)	79 (73)	78 (76)	35 (65)	66 (71)	52 (70)
Any ILD	174 (15)	103 (16)	10 (12)	26 (24)	20 (19)	1 (2)	7 (8)	7 (10)
Sarcoidosis	109 (9)	90 (14)	4 (5)	3 (3)	5 (5)	0	3 (3)	4 (5)
Pulmonary fibrosis	54 (5)	7 (1)	5 (6)	22 (20)	15 (15)	0	4 (4)	1 (1)
Remaining ILD	20 (2)	11 (2)	1 (1)	2 (2)	3 (3)	1 (2)	0	2 (3)
Any non-ILD lung disease	750 (64)	395 (61)	59 (68)	72 (66)	72 (70)	35 (65)	65 (70)	52 (70)
Asthma	253 (22)	160 (25)	21 (24)	17 (16)	9 (9)	7 (13)	18 (19)	21 (28)
Bronchitis	177 (15)	101 (16)	19 (22)	8 (7)	8 (8)	9 (17)	16 (17)	16 (22)
COPD	120 (10)	65 (10)	16 (18)	8 (7)	12 (12)	5 (9)	10 (11)	4 (5)
Emphysema	94 (8)	45 (7)	8 (9)	11 (10)	12 (12)	6 (11)	6 (7)	6 (8)
Pneumonia	82 (7)	35 (5)	9 (10)	11 (10)	10 (10)	3 (6)	7 (8)	7 (10)
Tuberculosis	114 (10)	56 (9)	8 (9)	10 (9)	16 (16)	7 (13)	9 (10)	8 (11)
Lung cancer	286 (25)	153 (24)	15 (17)	39 (36)	24 (23)	12 (22)	25 (27)	18 (24)
Lung disease n.s	186 (16)	85 (13)	15 (17)	20 (18)	25 (24)	12 (22)	14 (15)	15 (20)

Sarc = sarcoidosis; HP = hypersensitivity pneumonitis; IPF = idiopathic pulmonary fibrosis; uPF = unclassifiable pulmonary fibrosis; non-IPF IIP = an idiopathic interstitial pneumonia, other than IPF; aidILD = autoimmune disease interstitial lung disease; oILD = other interstitial lung disease; n.s. = not specified

### ILD in relatives

In total 174 patients (15%) reported the presence of ILD in their family, primarily consisting of 109 reports of sarcoidosis and 54 reports of pulmonary fibrosis (Table 2). There were 20 patients reporting ILDs other than sarcoidosis and pulmonary fibrosis, which included those related to occupational exposures (asbestosis, pneumoconiosis), hypersensitivity pneumonitis, and other rare ILDs (e.g. eosinophil pneumonia); denoted remaining ILD. Presence of ILD in the family varied widely between the diagnosis groups (range 2–24%). A family health history positive for any ILD was most frequently reported by IPF patients (24%; OR 2.6, CI 1.6–4.4), which was primarily due to 20% of IPF families with pulmonary fibrosis (OR 9.2, CI 4.7–17.9). Pulmonary fibrosis was also frequently reported (15%) by patients with uPF (OR 4.1, CI 2.0–8.2). Significant absence (1 report) of ILD in the family was observed for patients with non-IPF IIP (OR 0.1, CI 0.0–0.8). Sarcoidosis was only overrepresented (14%) in families of patients with sarcoidosis (OR 3.3, CI 1.9–5.8); in contrast to all other diagnosis groups where 0–5% of the patients reported sarcoidosis in their family. The sarcoidosis patients scarcely reported pulmonary fibrosis in their family (1%; OR 0.1, 95% CI 0.0–0.2). Table 3 shows significantly increased and decreased frequency of reported disease in the family.

**Table 3:** Association between patient diagnosis groups and self-reported disease in the family

Patient diagnosis group		Self-reported disease in family		Unadjusted	Adjusted for age	Adjusted for sex and age	
		*Significantly higher frequency of disease in the family*					
IPF		any ILD		1.9 (1.2–3.1)	2.6 (1.5–4.3)	2.6 (1.6–4.4)	
Sarcoidosis		sarcoidosis		4.3 (2.6–7.1)	3.3 (1.9–5.8)	3.3 (1.9–5.8)	
IPF		pulmonary fibrosis		8.1 (4.5–14.5)	7.9 (4.1–15.1)	9.2 (4.7–17.9)	
uPF		pulmonary fibrosis		4.5 (2.4–8.4)	3.9 (1.9–7.9)	4.1 (2.0–8.2)	
HP		COPD		2.1 (1.2–3.8)	2.4 (1.3–4.2)	2.3 (1.3–4.2)	
IPF		lung cancer		1.8 (1.2–2.8)	2.2 (1.4–3.4)	2.3 (1.4–3.5)	
		*Significantly lower frequency of disease in the family*					
Non-IPF IIP		any ILD		0.1 (0.0–0.7)	0.1 (0.0–0.8)	0.1 (0.0–0.8)	
Sarcoidosis		pulmonary fibrosis		0.1 (0.1–0.2)	0.1 (0.0–0.2)	0.1 (0.0–0.2)	
Sarcoidosis		non-ILD lung disease		0.7 (0.6–0.9)	0.6 (0.5–0.8)	0.6 (0.5–0.8)	
Sarcoidosis		lung disease, n.s		0.6 (0.5–0.9)	0.6 (0.4–0.9)	0.6 (0.4–0.9)	

For significant results (*p* < 0.05 Chi squared or Fisher’s exact when appropriate) the odds ratio and 95% confidence interval from logistic regression analysis (with and without adjustment for sex or age) are presented. HP = hypersensitivity pneumonitis; IPF = idiopathic pulmonary fibrosis; uPF = unclassifiable pulmonary fibrosis; non-IPF IIP = idiopathic interstitial pneumonia other than IPF; aidILD = autoimmune disease interstitial lung disease; oILD = other interstitial lung disease; n.s. = not specified

### Non-ILD lung disease in relatives

In 750 families (64%) of the ILD patients relatives with non-ILD lung diseases were reported. A positive family history for non-ILD lung disease was reported frequently across all diagnosis groups (range 61–70%, Table 2). However, specific associations between ILD diagnosis groups and reports of non-ILD lung disease in their family were found, and several of these remained significant after adjustment for sex and age of the patients (Table 3). HP patients reported a relative with COPD more frequently than patients from the other diagnosis groups (18%; OR 2.3, 95% CI 1.3–4.2), whilst lung cancer was most frequently reported by IPF patients (36%; OR 2.3, 95% CI 1.4–3.5).

### Familial ILD

We studied in further detail the number of ILD patients who reported any first degree relative with ILD, any other than first degree relative with ILD, or only other than first degree relatives (Table 4). Familial ILD was most frequently reported in first degree relatives of patients with ILD (72%), however reports of ILD in a second a more degree relative were also frequent (40%). This was most common in familial sarcoidosis where 60% of patients reported sarcoidosis in a first degree relative and 40% only in a second or more degree relative. By contrast, 86% of IPF and 80% of uPF patients reported a first degree relative with pulmonary fibrosis, whilst respectively 14 and 20% reported only other relatives. Within familial ILD (total n = 174), the reported diagnosis of relatives was strongly associated with the diagnosis of the patients. Out of 109 patients reporting relatives with sarcoidosis, 83% had received a diagnosis of sarcoidosis. Out of 54 patients reporting relatives with pulmonary fibrosis, 69% was diagnosed with IPF or uPF (Table 4).

**Table 4:** Reported relative types in familial ILD

	All	Sarc	HP	IPF	uPF	non-IPF IIP	aidILD	oILD
Any ILD, n (%)	174	103	10	26	20	1	7	7
1st degree relative	125 (72)	64 (62)	9 (90)	23 (88)	17 (85)	1 (100)	6 (86)	5 (71)
Other relative	70 (40)	52 (50)	2 (20)	7 (27)	6 (30)	0	1 (14)	2 (29)
Only other relative	49 (28)	39 (38)	1 (10)	3 (12)	3 (15)	0	1 (14)	2 (29)
Sarcoidosis	109	90	4	3	5	0	3	4
1st degree relative	70 (64)	54 (60)	3 (75)	3 (100)	5 (100)	0	3 (100)	2 (50)
Other relative	49 (45)	46 (51)	1 (25)	0	0	0	0	2 (50)
Only other relative	39 (36)	36 (40)	1 (25)	0	0	0	0	2 (50)
Pulmonary fibrosis	54	7	5	22	15	0	4	1
1st degree relative	45 (83)	5 (71)	5 (100)	19 (86)	12 (80)	0	3 (75)	1 (100)
Other relative	15 (28)	2 (29)	1 (20)	7 (32)	4 (27)	0	1 (25)	0
Only other relative	9 (17)	2 (29)	0	3 (14)	3 (20)	0	1 (25)	0
Remaining ILD	20	11	1	2	3	1	0	2
1st degree	14 (70)	7 (64)	1 (100)	2 (100)	1 (33)	1 (100)	0	2 (100)
Other relative	7 (35)	5 (45)	0	0	2 (67)	0	0	0
Only other relative	6 (30)	4 (36)	0	0	2 (67)	0	0	0

HP = hypersensitivity pneumonitis; IPF = idiopathic pulmonary fibrosis; uPF = unclassifiable pulmonary fibrosis; non-IPF IIP = idiopathic interstitial pneumonia other than IPF; aidILD = autoimmune ILD; oILD = other ILD. Familial disease is an ILD patient reporting one or more relatives with an ILD

**Fig. 2:**
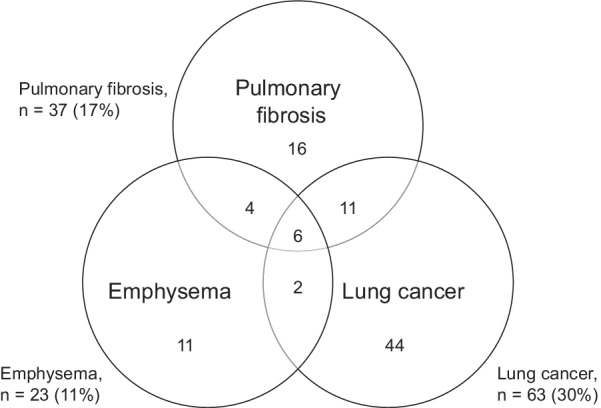
Presence of pulmonary fibrosis, emphysema and lung cancer in relatives of 212 patients (100%) with pulmonary fibrosis. Overlapping area’s represent patients reporting both diseases in the family

We investigated if specific reports of non-ILD lung disease differed between familial and sporadic ILD patients. For sarcoidosis no differences were found. In this ILD population, pulmonary fibrosis in relatives was predominantly reported by IPF and uPF patients (Table 2). To investigate differences in pulmonary fibrosis, we combined IPF and uPF data to form a group with familial PF (FPF) and a group with sporadic PF (SPF). Next, we compared the frequency of reported non-ILD lung disease between FPF and SPF patient groups (Table 5). Patients with self-reported FPF were more likely to report emphysema (n = 10, 27%; OR 4.6, CI 1.8–11.6) and lung cancer (n = 17, 46%; OR 2.4, CI 1.2–4.9) in their family (Table 5). Figure 2 presents the total numbers of patients with pulmonary fibrosis who reported pulmonary fibrosis, emphysema, or lung cancer in their family. Out of 37 FPF patients 21 (57%) reported emphysema and/or lung cancer in relatives, versus 57 out of 175 patients (33%) with sporadic disease. Furthermore, 6 out of 37 patients with FPF, reported both emphysema and lung cancer versus 2 out of 175 with SPF (16% FPF versus 1% SPF: OR 16.7 (95% CI 3.2–86.8)).

**Table 5:** Comparison of reported lung disease in the family between patients with sporadic and familial sarcoidosis or pulmonary fibrosis

	Sarcoidosis		pulmonary fibrosis	
	Sporadic	Familial	Sporadic	Familial
	n = 554	n = 90	n = 175	n = 37
*Disease in relatives, n (%):*				
Sarcoidosis	0 (0)	90 (100)	7 (4)	1 (3)
Pulmonary fibrosis	6 (1)	1 (1)	0 (0)	37 (100)
Remaining ILD	8 (1)	3 (3)	3 (2)	2 (5)
Non-ILD lung disease	337 (61)	58 (64)	118 (67)	26 (70)
Asthma	136 (25)	24 (27)	21 (12)	5 (14)
Bronchitis	84 (15)	17 (19)	15 (9)	1 (3)
COPD	55 (10)	10 (11)	16 (9)	4 (11)
Emphysema	38 (7)	7 (8)	13 (7)	10 (27)* P 0.002 OR 4.6 (1.8–11.6)
Pneumonia	33 (6)	2 (2)	19 (11)	2 (5)
Tuberculosis	49 (9)	7 (8)	21 (12)	5 (14)
Lung cancer	127 (23)	26 (29)	46 (26)	17 (46)* P 0.017 OR 2.4 (1.2–4.9)
Lung disease n.s	70 (13)	15 (17)	37 (21)	8 (22)

*Significant difference (Chi squared or Fisher’s exact, *p* < 0.05) between sporadic and familial pulmonary fibrosis. The odds ratio (OR) with 95% confidence interval between brackets are presented in case of significant results. n.s. = not specified

## Discussion

In this study we found clustering patterns of ILD and non-ILD lung disease in diagnosis groups of ILD patients. ILD and non ILD-lung disease was reported in 15% and 64% of ILD patients’ relatives respectively. Reports on relatives with the same ILD as the patient were frequent in IPF, uPF, and sarcoidosis. Reporting relatives with emphysema and lung cancer was a strong predictor for familial pulmonary fibrosis, in our study.

The frequency of familial occurrence in several ILD diagnoses was previously reported by Cutting [16] who reported 25% in IPF, 7.7% in CTD-ILD and 14% in chronic HP. Although there were some differences in grouping of ILD patients, these numbers are remarkably similar to our findings (Table 2). The most pronounced congruence between their and our findings is the highest frequency of FPF in IPF patients’ families (25% Cutting vs 20% current study). Strikingly, the non-IPF IIP (diagnosis types within these group are presented in Additional file 2: Table 1) patients in our population did not report any relative with either sarcoidosis or pulmonary fibrosis. Krauss and colleagues [15] also found a lower prevalence of FPF among patients with other IIPs than IPF. In the current classification of ILD, IPF is a diagnosis within the group of IIP [25]. In several studies on familial IIP, non-IPF IIP was included in the population and this often yielded positive results [17, 19, 31–33]. In our study, however, we separated the classifiable non-IPF IIP from the unclassifiable pulmonary fibrosis patients, which revealed possibly unique characteristics of the non-IPF IIP group. This group was mainly (90%) composed of patients with a diagnosis of (cryptogenic) organizing pneumonia (COP), non-specific interstitial pneumonia, and smoking related ILD (respiratory bronchiolitis-ILD and desquamative interstitial pneumonia) (Additional file 2: Table 1). COP and these smoking related ILDs are not among the so-called progressive-fibrosing interstitial lung diseases and often respond to removal of the disease trigger or immunosuppression [25, 28]. These findings together suggest that the ILD patients with a positive family history for pulmonary fibrosis, are those with a progressive-fibrosing ILD, in which IPF predominates.

Determining presence of disease in families of patients with pulmonary fibrosis is important to identify patients with suspected monogenic pulmonary fibrosis [2, 3]. However, it is unclear what the definition of familial ILD should involve and familial disease estimates for ILD have varied widely, presumably at least in part because of the lack of a consensus definition [1]. The usefulness of family health history data also depends on how the data are collected, which is not standardized across many clinical practices. In our study, we found that in familial pulmonary fibrosis 14–20% consist only of reports for second or more degree relatives with pulmonary fibrosis. When restricting the definition of familial pulmonary fibrosis to first degree relatives only, 3% of the IPF and uPF patients (3 out of 109 IPF and 103 uPF patients) with familial disease would be missed. It is furthermore important to realize that disease may skip a generation, when there is a reduced penetrance [15, 34]. In the sarcoidosis group, 40% of familial sarcoidosis consist of reports for second or more degree relatives only. For the entire sarcoidosis population, 6% of patients with familial disease (36 out of 644) would be missed, when restricting its definition to first degree relatives only. To date it is unclear if inclusion of all forms of ILD in the definition of familial ILD has clinical significance when trying to identify subjects at risk for developing progressive pulmonary fibrosis. When including sarcoidosis and other remaining ILD in the definition of familial disease in patients with IPF or uPF, its frequency would be respectively 4% and 5% higher (Table 2). For sarcoidosis, there would be an extra 2% of patients with familial ILD (Table 2).

We previously reviewed the literature on the association between sarcoidosis and immune-mediated (chronic inflammatory or autoimmune) diseases in patients and families [11]. In multiple studies an increased frequency of sarcoidosis in patients and family members of patients with immune-mediated diseases was found [11]. In our current study however, the aidILD patient group did not report sarcoidosis more frequently in their family. This finding suggests that patients with autoimmune disease who develop ILD may not represent the general population of patients with immune-mediated disease. The current study shows no clustering of pulmonary fibrosis with sarcoidosis, furthermore none of the associations with non-ILD lung diseases overlap between pulmonary fibrosis and sarcoidosis. A clinical follow-up study would be needed to investigate if prognostic differences between patients with different combinations of familial ILD exist.

Among the older patients in our population were those with IPF and HP, and there was no significant difference in mean age between these ILD diagnosis groups (Table 1). There was however, a striking difference between these groups of patients in reporting the aging lung diseases lung cancer, COPD and emphysema. IPF patients reported relatives with lung cancer exceedingly frequent (36%, OR 2.3; CI 1.4–3.5; Table 3); whilst those with HP reported only 17%, which was the least, even less than sarcoidosis patients who were youngest (Table 2). COPD on the contrary was most reported by the patients with HP (18%, OR 2.3; CI 1.3–4.2; Table 3). These findings remained significant after adjustment for age and sex of the reporting patients and may indicate shared etiologic factors in HP and COPD on the one hand and IPF and lung cancer on the other hand. Presence of shared and opposite risk alleles for disease [35], or differences in the respiratory microbiome [36] may direct disease phenotype in the aging lung.

Next to the increased overlap of HP and COPD in families, we found that familial pulmonary fibrosis (FPF) clusters with emphysema and with lung cancer. This indicates that a shared driver for FPF, emphysema, and lung cancer may be present. The novelty of our findings is that the differences concern not the individual patients, but their families. Environmental drivers for these diseases are known to overlap, and include most importantly exposure to cigarette smoke. Although we have no data on exposure in our study, in other studies the majority of patients with pulmonary fibrosis, including those with familial disease, have a positive smoking history [17, 19, 24, 37–39]. However, the amount of ever smokers in another Dutch cohort of pulmonary fibrosis patients was compared to patients with SPF lower in FPF [40]. Evidence for an important role of genetic risk factors in the occurrence of pulmonary fibrosis is furthermore well established [1, 41]. The role of environmental factors, such as active and passive smoking, therefore deserves further investigation, particularly in familial disease. Future research, actively involving family members, may reveal if there is an association between familial reports of pulmonary fibrosis, emphysema, lung cancer and smoking in families, and if and how this interacts with known genetic risk factors. Overlapping pathways between pulmonary fibrosis and cancer have been suggested before. Cancer markers CA-19, CA-125, and CA15-3 have been identified as prognostic biomarkers in IPF [42, 43]. In this population, there is a subgroup of patients with surfactant related genetic pulmonary fibrosis or short telomere syndromes, which are both associated with a predisposition to cancer and emphysematous changes [2, 3, 13]. The prevalence of surfactant and telomere related gene mutations was found to be respectively 3–8% and 25–36% in European populations of patients with possible genetic pulmonary fibrosis [2, 3]. Recently, it was shown that screened asymptomatic first degree relatives of patients with sporadic and familial pulmonary fibrosis had similar increased risk for interstitial lung abnormalities and a diagnosis of ILD [21]. In this study, the definition of FPF consisted of the presence of at least one other person with ILD in a five generations pedigree; sporadic IPF was defined by families where the proband was the only known case of ILD in the pedigree. Although numbers were small, these data indicate that patients and relatives at risk for familial or genetic pulmonary fibrosis are not limited to those with a positive family history of ILD. While multiple reasons for a negative family history for ILD exist, such as being the first in your family with ILD, absence of sibs, reduced penetrance, etc., our study shows that information on non-ILD lung diseases in relatives may aid identification of at-risk subjects. Emphysema and lung cancer are common diseases, but in this study only 1% of sporadic PF patients report both diseases in the family, in comparison to 16% of FPF patients (OR 16.7; CI 3.2–86.8).

We acknowledge that our study had limitations which may have influenced the results. The study is based on self-reported disease in relatives of patients, without confirmation of the reported diseases. It is likely that patients who are treated at the hospital’s respiratory department are more aware of lung diseases among their relatives. However, this would be true for all patient diagnostic groups and may not explain the differences between them. Another limitation is that we do not know the size of the families, which is relevant to the chance of the occurrence of disease in a family. Also, this is a retrospective study, and unfortunately there were missing data on informative population characteristics, such as ethnicity. For ILD, it is known that ethnicity associates with risk for disease and outcome [44]. IPF is predominantly found in White and less in Black persons [45]. From previous studies we know that our Dutch cohort of IPF and FPF patients consists of patients of mostly European and some Asian descent, which is in line with other studies investigating familial disease [18, 32]. A strength of the study involves the structured questionnaire that was completed prior to the first visit of the patients to the clinic (Additional file 1).

In conclusion, we aimed to identify differences between diagnosis groups of ILD patients in self-reported disease clustering of ILD and non-ILD lung disease in their relatives.

We found that the frequency of familial ILD varied widely between diagnosis groups. Reporting familial ILD was highly predictive of the patient’s diagnosis; and reporting emphysema and lung cancer specifically of FPF presence. Shared pathogenesis may underlie clustering of ILD and non-ILD lung diseases and provide rationale for future research.

## Supplementary Information

**Additional file 1.** Questionnaire on family health history.

**Additional file 2.** Diagnosis types within patient diagnosis groups.

## Data Availability

All data analysed during this study are included in this article.

## Abbreviations

ILD: Interstitial lung disease
Sarc: Sarcoidosis
HP: Hypersensitivity pneumonitis
IPF: Idiopathic pulmonary fibrosis
uPF: Unclassifiable pulmonary fibrosis
non-IPF IIP: An idiopathic interstitial pneumonia, other than IPF
aidILD: Autoimmune disease interstitial lung disease
oILD: Other interstitial lung disease
SPF: Sporadic pulmonary fibrosis
FPF: Familial pulmonary fibrosis

## Declarations

### In accordance statement

The research was conducted in accordance with relevant guidelines and regulations.
