# Supplementary material for: Clustering of lung diseases in the family of interstitial lung disease patients
Source: BMC Pulm Med. 2022 Apr 7;22:134. doi: 10.1186/s12890-022-01927-x (PMC8991662; doi:10.1186/s12890-022-01927-x)
Supplement: Supplementary file 1 — Additional file 1. Questionnaire on family health history. [file 12890_2022_1927_MOESM1_ESM.docx]

Supplementary file 1. Questionnaire on family health history¹̓².

|  | Questions |
| --- | --- |
| Front page |  |
|  | Date of questionnaire completion  Data about yourself  (first and last name, date and place of birth, types of disease, age at diagnosis, hospital where diagnosed) |
| Page 2 |  |
|  | Data about your parents and grandparents  (date and place of birth, types of disease, age at diagnosis, hospital where diagnosed, if died at which date) |
| Page 3 |  |
|  | Data about your children  (date of birth, sex, types of disease, age at diagnosis, hospital where diagnosed, if died at which date, if children sex and number) |
| Page 4 |  |
|  | Data about your brothers and sisters  (date of birth, sex, types of disease, age at diagnosis, hospital where diagnosed, if died at which date, if children sex and number) |
| Page 5 |  |
|  | Data about your half-brothers and half-sisters  (date of birth, sex, types of disease, age at diagnosis, hospital where diagnosed, if died at which date, if children sex and number) |
| Page 6 |  |
|  | Data about your fathers’ brothers and sisters, that is your uncles and aunts  (date of birth, sex, types of disease, age at diagnosis, hospital where diagnosed, if died at which date, if children sex and number) |
| Page 7 |  |
|  | Data about your mothers’ brothers and sisters, that is your uncles and aunts  (date of birth, sex, types of disease, age at diagnosis, hospital where diagnosed, if died at which date, if children sex and number) |
| ³Page 8 |  |
|  | Data about other relatives  (date of birth, place of birth, sex, types of disease, age at diagnosis, hospital where diagnosed, if died at which date and all of these questions for the parents of this specific relative as well) |

¹The questionnaire was not included as a file, because it is in Dutch, and contains confidential patient information. ²This is a retrospective study and not all patients answered all questions. The data from some questions were too scarce to allow any evaluation or data analysis. ³The majority of the patients reported only nephews and nieces here. In this study we thus included data on disease in parents, grandparents, children, siblings, uncles and aunts, nephews and nieces.
