# Supplementary material for: Clustering of lung diseases in the family of interstitial lung disease patients
Source: BMC Pulm Med. 2022 Apr 7;22:134. doi: 10.1186/s12890-022-01927-x (PMC8991662; doi:10.1186/s12890-022-01927-x)
Supplement: Supplementary file 2 — Additional file 2. Diagnosis types within patient diagnosis groups. [file 12890_2022_1927_MOESM2_ESM.docx]

Supplementary table 1. Diagnosis types within patient diagnosis groups. ¹

| Diagnosis group | Diagnosis type | n(%) |
| --- | --- | --- |
|  |  |  |
| Idiopathic interstitial pneumonia, other than idiopathic pulmonary fibrosis |  |  |
|  | (cryptogenic) organising pneumonia | 16(30) |
|  | acute fibrinous organising pneumonia | 1(2) |
|  | lymphoid interstitial pneumonia | 1(2) |
|  | non-specific interstitial pneumonia | 11(20) |
|  | pleuroparenchymal fibroelastosis | 3(6) |
| smoking related |  |  |
|  | desquamative interstitial pneumonia | 12(22) |
|  | respiratory bronchiolitis intersititial lung disease | 10(19) |
| Autoimmune disease interstitial lung disease |  |  |
|  | ankylosing spondylitis | 1(1) |
|  | mixed connective tissue disease | 1(1) |
|  | polymyositis-dermatomyositis | 13(14) |
|  | rheumatoid arthritis | 16(17) |
|  | scleroderma | 9(10) |
|  | Sjögren’s syndrome | 8(9) |
|  | unclassified connective tissue disease | 4(4) |
|  | IG4 related disease | 2(2) |
|  | interstitial pneumonia with autoimmune features | 22(24) |
|  | pulmonary alveolar proteinosis | 12(13) |
|  | vasculitis | 5(5) |
| Other interstitial lung disease |  |  |
|  | amyloidosis | 2(3) |
|  | asbestosis | 5(7) |
|  | drug-induced interstitial lung disease | 18(24) |
|  | eosinophil pneumonia | 5(7) |
|  | idiopathic dendritic pulmonary ossification | 1(1) |
|  | lymphangioleiomyomatosis | 23(31) |
|  | not specified exposure related interstitial lung disease | 3(4) |
|  | pneumoconiosis | 5(7) |
|  | pulmonary Langerhans’cell histiocytosis | 7(9) |
|  | pulmonary lymphoma | 1(1) |
|  | unclassified interstitial lung disease | 4(5) |

¹Within the diagnosis groups sarcoidosis, hypersensitivity pneumonitis, idiopathic pulmonary fibrosis, and unclassifiable pulmonary fibrosis there were no diagnosis subtypes.
